# Detection of mild cognitive impairment in a community‐dwelling population using quantitative, multiparametric MRI‐based classification

**DOI:** 10.1002/hbm.24554

**Published:** 2019-02-25

**Authors:** Mark J. R. J. Bouts, Jeroen van der Grond, Meike W. Vernooij, Marisa Koini, Tijn M. Schouten, Frank de Vos, Rogier A. Feis, Lotte G. M. Cremers, Anita Lechner, Reinhold Schmidt, Mark de Rooij, Wiro J. Niessen, M. Arfan Ikram, Serge A. R. B. Rombouts

**Affiliations:** ^1^ Institute of Psychology Leiden University Leiden the Netherlands; ^2^ Department of Radiology Leiden University Medical Center Leiden the Netherlands; ^3^ Leiden Institute for Brain and Cognition Leiden University Leiden the Netherlands; ^4^ Department of Epidemiology Erasmus MC University Medical Center Rotterdam the Netherlands; ^5^ Department of Radiology and Nuclear Medicine Erasmus MC University Medical Center Rotterdam the Netherlands; ^6^ Department of Neurology Medical University of Graz Austria; ^7^ Department of Medical Informatics Erasmus MC University Medical Center Rotterdam the Netherlands; ^8^ Faculty of Applied Sciences Delft University of Technology Delft the Netherlands; ^9^ Department of Neurology Erasmus MC University Medical Center Rotterdam the Netherlands

**Keywords:** Alzheimer's disease, classification, community‐dwelling cohort, diffusion tensor imaging, machine learning, mild cognitive impairment, MRI

## Abstract

Early and accurate mild cognitive impairment (MCI) detection within a heterogeneous, nonclinical population is needed to improve care for persons at risk of developing dementia. Magnetic resonance imaging (MRI)‐based classification may aid early diagnosis of MCI, but has only been applied within clinical cohorts. We aimed to determine the generalizability of MRI‐based classification probability scores to detect MCI on an individual basis within a general population. To determine classification probability scores, an AD, mild‐AD, and moderate‐AD detection model were created with anatomical and diffusion MRI measures calculated from a clinical Alzheimer's Disease (AD) cohort and subsequently applied to a population‐based cohort with 48 MCI and 617 normal aging subjects. Each model's ability to detect MCI was quantified using area under the receiver operating characteristic curve (AUC) and compared with an MCI detection model trained and applied to the population‐based cohort. The AD‐model and mild‐AD identified MCI from controls better than chance level (AUC = 0.600, *p* = 0.025; AUC = 0.619, *p* = 0.008). In contrast, the moderate‐AD‐model was not able to separate MCI from normal aging (AUC = 0.567, *p* = 0.147). The MCI‐model was able to separate MCI from controls better than chance (*p* = 0.014) with mean AUC values comparable with the AD‐model (AUC = 0.611, *p* = 1.0). Within our population‐based cohort, classification models detected MCI better than chance. Nevertheless, classification performance rates were moderate and may be insufficient to facilitate robust MRI‐based MCI detection on an individual basis. Our data indicate that multiparametric MRI‐based classification algorithms, that are effective in clinical cohorts, may not straightforwardly translate to applications in a general population.

## 1 INTRODUCTION

Alzheimer's disease (AD) is a progressive neurodegenerative disorder with a substantial personal and increasing societal impact (Alzheimer's Association, 2018; Hurd, Martorell, Delavande, Mullen, & Langa, 2013). Early and accurate diagnosis of AD is imperative for adequate patient management, improved personalized care, and continued development of effective disease‐modifying therapies (Alzheimer's Association, 2018; Bachurin, Gavrilova, Samsonova, Barreto, & Aliev, 2018; Petersen, 2011). Mild cognitive impairment (MCI) is a transitional stage where cognitive impairments are in between normal aging and very early dementia (Petersen, 2016). Individuals with MCI are more likely to convert to dementia with an annual rate of 5–10% compared with 1–2% within the general population (Petersen, 2011). Identifying individuals with MCI offers unique opportunities to facilitate and improve interventions that are more likely to be effective (Alzheimer's Association, 2018; Bachurin et al., 2018). Yet, reliable MCI diagnoses are often difficult to achieve. Cognitive manifestations are subtle, heterogeneous, and regularly remain unnoticed, especially in high functioning individuals that are capable of maintaining normal levels of functioning despite demonstrating overt cognitive impairment (Petersen, 2011, 2016).

Complementary to cognitive determinants, magnetic resonance imaging (MRI) has shown in defined clinical populations to provide valuable insights that corroborate MCI diagnosis (Buckner, 2004; Fan, Batmanghelich, Clark, & Davatzikos, 2008; Petersen, 2016) and aid in the prediction of subsequent progression to dementia (McEvoy et al., 2011; Misra, Fan, & Davatzikos, 2009; Tapiola et al., 2008). MRI has revealed specific structural differences that include the extent and location of gray matter (GM) atrophy (Tapiola et al., 2008; Wang et al., 2017) and variations in diffusion tensor imaging (DTI) measures within the white matter (WM) (De Bruijn et al., 2014; Wang et al., 2017; Zhuang et al., 2010). These GM atrophy and WM DTI measure values are in between those of controls and dementia and may even precede cognitive deficits (Buckner, 2004; Fan et al., 2008).

In order to contribute to diagnostic standards, MRI‐derived biomarkers should be able to reliably identify MCI subjects on an individual level. To this end, imaging‐derived markers have been used in the development of MRI‐based classification algorithms. These algorithms integrate various MRI measures within a single, quantitative probabilistic score in order to, on an individual basis, differentiate patients from cognitively normal controls (Bouts et al., 2018; Cuingnet et al., 2011; de Vos et al., 2016; Dyrba et al., 2015; Misra et al., 2009; Rathore, Habes, Iftikhar, Shacklett, & Davatzikos, 2017; Schouten et al., 2016; Schouten et al., 2017; Wee et al., 2011) and identify those MCI subjects most likely to progress to dementia (Eskildsen et al., 2013; Misra et al., 2009). This probabilistic score may also serve as a surrogate measure of disease severity on a continuum from cognitively normal to dementia, with MCI being represented by intermediate scores (Adaszewski, Dukart, Kherif, Frackowiak, & Draganski, 2013; Eskildsen et al., 2013). Nevertheless, these algorithms are mostly evaluated on relatively small, carefully selected, clinical cohorts. It remains to be elucidated how well these detection models translate to general populations where disease induced manifestations are likely to be less conspicuous and heterogeneous across subjects (Dukart, Schroeter, & Mueller, 2011; Misra et al., 2009; Murray et al., 2011; Rathore et al., 2017), disease (sub)types (Adaszewski et al., 2013; Dong et al., 2017; Eskildsen et al., 2013), and time to conversion (Adaszewski et al., 2013; Dong et al., 2017; Eskildsen et al., 2013). Detection within these nonclinical populations should also be reliable in order to improve patient diagnostic standards, improve patient selection for clinical trials, and facilitate tailored early stage intervention.

In this study we aimed to determine the generalizability of MRI‐based classification probability scores to detect MCI on an individual basis within a general population. To determine classification probability scores, we used a clinically defined AD cohort to train an AD‐, a mild‐AD, and a moderate‐AD classification model and subsequently applied these models to a community‐dwelling cohort to determine each model's ability to detect MCI from normal aging. Each model's classification performance was subsequently compared with an MCI classification model trained and applied to the community‐dwelling cohort.

## 2 MATERIALS AND METHODS

This study involved a retrospective analysis of previously published data (De Bruijn et al., 2014; Schouten et al., 2016) acquired at two different centers. All data were collected in accordance with regional research regulations, were approved by the local ethics committees, and conformed to the Declaration of Helsinki.

### 2.1 Design

To determine the ability to detect MCI from normal aging within a community‐dwelling cohort, we employed four MRI‐based probabilistic classification models. This first model was recently introduced and validated in two separate clinical cohorts (Bouts et al., 2018; Schouten et al., 2016). We trained this model with AD patients and control subjects of a separate clinical AD cohort. This model, hereafter referenced as AD‐model, included subjects of a wider AD spectrum (mild and moderate AD patients) and was used to determine whether probability scores of a model trained for AD classification are able to identify MCI from normal aging subjects in a nonclinical cohort. The second and third model were trained using sub‐populations of the clinical AD cohort. These models were created to further disentangle classification performance of the AD‐model in relation to symptom severity. One model was trained with subjects with relatively mild AD symptoms (i.e., mini‐mental state examination score [MMSE] > 20; Schouten et al., 2016). This model, hereafter referenced as mild‐AD‐model, was used to determine the influence of less pronounced AD signatures on MCI detection performance in the community‐dwelling cohort. The other model was trained with more moderate AD subjects (MMSE ≤ 20) to determine the influence of more advanced AD signatures on MCI detection performance in the community‐dwelling cohort. Finally, a fourth model was trained with MCI and control subjects of the community‐dwelling cohort. This model, hereafter referred to as MCI‐model, was created to contextualize the classification performance results obtained with the previous models. All MRI‐processing, feature selection, and classification procedures were identical for all models.

### 2.2 Participants

Subjects of the Rotterdam study were used to create the community‐dwelling cohort (hereafter: RS cohort). The Rotterdam study is a prospective population‐based cohort study in which inhabitants of the well‐defined Ommoord district in Rotterdam, the Netherlands, participate upon invitation. Study details can be found elsewhere (Ikram et al., 2015; Ikram et al., 2017). For the present analysis we selected 682 subjects that were older than 60 years of age, underwent MRI in the period 2002–2005, did not have MRI‐defined cortical infarcts, and had data available for MCI diagnosis (De Bruijn et al., 2014). Subjects were diagnosed as MCI according to criteria previously derived for the Rotterdam study (Adams et al., 2015; De Bruijn et al., 2014). In brief, participants were considered MCI when the following criteria were met: (a) presence of subjective cognitive complaints, (b) presence of objective cognitive impairment, and (c) absence of dementia. Subjective memory complaints were evaluated per interview. At least one affirmative answer to questions on memory or daily functioning resulted in a subject complaint positive status. Objective cognitive impairment was determined using a cognitive test battery that comprised of letter‐digit substitution task, Stroop test, verbal fluency test, and 15‐word verbal learning test based on Rey's recall of words (De Bruijn et al., 2014). Scores were summarized by compound scores for various cognitive domains including memory function, information‐processing speed, and executive function (De Bruijn et al., 2014). Subjects were classified as objectively cognitively impaired when they scored 1.5 standard deviation (SD) lower than the age and education adjusted means of the study population. Individuals with MCI who had impaired test scores on memory function (irrespective of other domains) were defined as amnestic MCI. MCI subjects having normal memory function, but impaired test scores on executive function or information‐processing speed were defined as nonamnestic MCI.

The AD‐, mild‐AD, and moderate‐AD‐model were trained using data from a separate clinical AD cohort which was previously described in more detail (Schouten et al., 2016). In brief, this cohort was acquired at the Medical University of Graz and included AD patients taken from the baseline data of the prospective registry on dementia (PRODEM; Seiler et al., 2012). Patients were diagnosed as AD according to DSM‐IV criteria (American Psychiatric Association, 2000) and NINCDS‐ADRDA criteria for AD diagnosis (McKhann et al., 1984). Control subjects were taken from the Austrian Stroke Prevention Study. These control subjects were scanned under similar settings as the AD patients, including the same MRI acquisition protocol, MRI scanner, and time period. For our analysis, we included 77 AD patients—39 AD patients had mild AD (MMSE>20), 38 AD patients with moderate AD (MMSE<=20; Perneczky et al., 2006) (Supporting Information Table S1)—who were between 47 and 83 in age, and 173 healthy, age‐matched controls (Table 1).

**Table 1:** Demographics of the AD and RS cohort

	AD cohort		RS cohort	
	Control	AD	Control	MCI
*N*	173	77	617	48
Age (mean ± *SD*)	66.1 ± 8.7	68.6 ± 8.6	67.3 ± 5.2	68.8 ± 6.6#
Female gender (%)	99 (57.2)	46 (59.7)	319 (51.7)	23 (47.9)
Disease duration (months)		26.4 ± 24.6		
MMSE (mean ± *SD*)	27.5 ± 1.8	20.4 ± 4.5**	28.1 ± 2.0	26.9 ± 1.8** ^,^ ##

AD: Alzheimer's disease, MCI: mild cognitive impairment, MMSE: mini‐mental state examination, *SD*: standard deviation.

** Versus control subjects, *p* < 0.01.

# Versus AD cohort, *p* < 0.05.

## Versus AD cohort, *p* < 0.001.

### 2.3 MRI processing

MRI protocols and MRI preprocessing procedures are described in more detail in the Supporting Information. All 682 RS subjects were scanned on a 1.5 T MRI scanner (GE Healthcare) with an 8‐channel head coil. The 250 subjects of the AD cohort were scanned on a 3 T MRI scanner (TrioTim, Siemens) with a 12‐channel head coil. Both protocols included a 3D isotropic T1‐weighted image and a diffusion MRI dataset with a maximum b‐value of 1,000 s/mm^2^. Preprocessing procedures of the 3DT_1_w images and diffusion MRI were similar for both cohorts and followed those previously described (Bouts et al., 2018). The processed maps were subsequently used for feature extraction. From the 3DT_1_w data, 96 cortical GM density (GMD), 14 deep GM volume (DGMV), and 20 average WM density (WMD) values were extracted per subject. Mean cortical values were calculated by weighting the regions of the cortical Harvard–Oxford (HO) probabilistic anatomical brain atlas by the regional probabilistic GM tissue segmentation. Feature values of the deep GM structures were calculated by normalizing volumes of the bilateral thalamus, caudate nucleus, putamen, globus pallidus, nucleus accumbens, amygdala, and hippocampi by the intracranial volume. The 20 tracts of probabilistic Johns‐Hopkins‐University (JHU) white‐matter tractography atlas were weighted by the tract‐specific probabilistic WM segmentation values to obtain values of WM density (WMD). These 20 JHU‐tracts were also used to extract tract‐weighted mean fractional anisotropy (FA) and mean diffusivity (MD) values from the DTI data after voxel‐wise projecting each value onto the standard FMRIB58_FA skeleton (Smith et al., 2007).

To compensate for nonbiological differences between cohorts that included MRI acquisition settings, head‐coil, and field strength discrepancies, we determined linear correction factors by repeatedly, randomly selecting a balanced set of 68 unique control subjects of the AD and RS cohorts to estimate a correction factor that was subsequently applied to those subjects not used in correction factor estimation (Adaszewski et al., 2013; Dukart et al., 2011). This process was repeated five times to make sure that all subjects' feature vectors were corrected.

### 2.4 Classification

The above described structural and diffusion features were subsequently used for classification analysis. For classification analysis we used elastic net regression, a previously successfully employed classifier for detection of AD (Bouts et al., 2018; de Vos et al., 2016; de Vos et al., 2017; Schouten et al., 2016; Schouten et al., 2017; Teipel et al., 2017), (presymptomatic) FTD (Bouts et al., 2018; Feis et al., 2018), or differentiation between these dementia‐types (Bouts et al., 2018). An elastic net regression model effectively selects only those features relevant for classification by estimating a sparse regression model that selects a subset of all provided features using feature selection and feature weight penalties during regression. Consequently, this provides a means to address the imbalance between the limited number of training subjects and the large number of training features (Zou & Hastie, 2005).

### 2.5 Cross‐validation

Model training and optimization procedures were in accordance with those detailed previously (Bouts et al., 2018; Schouten et al., 2016). In brief, after each feature was standardized to zero mean and unit variance, either single or all MRI measures (i.e., GMD, DGMV, WMD, FA, or MD) derived from the training data were alternately used to train a classification model using nested 10‐fold cross‐validation. Cross‐validation aids in determining the optimal set of operational parameters and overall classification performance without introducing bias by using the same subject for training and testing (Kriegeskorte, Simmons, Bellgowan, & Baker, 2009; Varma & Simon, 2006). The data is iteratively subdivided in separate test and training sets and used in two, nested cross‐validation loops. The outer loop was used to determine the overall classification performance, the inner loop further subdivided the training data to determine the best operational parameters for the penalty terms without overestimating classification performance (Varma & Simon, 2006; Varoquaux et al., 2017). This process was repeated 10 times to ascertain that each subject was part of the test set of the outer loop exactly once. The entire cross‐validation procedure was repeated 100 times to reduce variance resulting from random partitioning in training and test folds, and to report the range of observed outcomes under different train and test conditions. Age and gender were included into all models without any penalty to ensure that estimated feature regression coefficients were conditional on subject age and gender.

### 2.6 AD‐model

Training of the AD‐model followed a specific procedure to assure that the most appropriate model was used for MCI detection comparison. First, repeated 10‐fold cross‐validation was used to determine whether an individual MRI measure or the combined set of measures attained highest classification performance within the AD cohort. The feature set that attained highest classification performance was then used to train the AD‐model using all AD patients and control subjects of the AD cohort. This AD‐model was then applied to the feature vector of each participant in the RS cohort to obtain an AD probability score ranging between 0 and 1, where 0 represented control and 1 AD subject. Translated to the RS cohort, this score indicated how similar a participant was to an AD patient. The procedure of calculating the center correction factor, training using AD cohort data, and testing on the RS cohort data was repeated 100 times to be consistent with the cross‐validation procedure.

### 2.7 Mild‐AD‐model

The mild‐AD‐model followed the same procedure as the AD model. However, for this model we considered only the mild‐AD patients and all controls of the AD cohort for training. Repeated 10‐fold cross validation determined whether a single MRI measure or combination of MRI measures attained highest classification performance for detecting mild‐AD symptoms within the AD cohort. The set of features that attained highest classification performance was subsequently used to train a mild‐AD model with all the mild‐AD patients and controls of the AD cohort. This mild‐AD‐model was then applied to the feature vector of each participant of the RS cohort to obtain an AD probability score ranging between 0 and 1. For this model, 0 represented a cognitively normal (i.e., control) subject whereas 1 represented a mild‐AD patient. Translated to the RS cohort, this score indicated how similar a participant was to an AD patient with relatively mild‐AD symptoms (i.e., MMSE>20). Again, the calculation of center correction factors, training using AD cohort data, and testing on the RS cohort data were repeated 100 times to be consistent with the cross‐validation procedure.

### 2.8 Moderate‐AD‐model

The moderate‐AD‐model followed the same procedure as the mild‐AD model. However, we only considered moderate‐AD patients and all controls of the AD cohort for training. Repeated 10‐fold cross validation determined whether a single MRI measure or combination of MRI measures attained highest classification performance for detecting moderate‐AD symptoms within the AD cohort. The set of features that attained highest classification performance was subsequently used to train a moderate‐AD model with all the moderate‐AD patients and controls of the AD cohort. This moderate‐AD‐model was then applied to the feature vector of each participant of the RS cohort to obtain an AD probability score ranging between 0 and 1. For this model, 0 represented a cognitively normal (i.e., control) subject whereas 1 represented a moderate‐AD patient. Translated to the RS cohort, this score indicated how similar a participant was to an AD patient with moderate‐AD symptoms (i.e., MMSE ≤ 20). The calculation of center correction factors, training using AD cohort data, and testing on the RS cohort data were repeated 100 times to be consistent with the cross‐validation procedure.

### 2.9 MCI‐model

For the MCI‐model, the model‐development procedure was limited to cross‐validation within the RS cohort. One‐hundred times repeated 10‐fold cross validation determined whether a single MRI measure or combination of MRI measures attained highest classification performance for detecting MCI within the RS cohort. The set of features that attained highest classification performance was used for MCI probability score calculation. MCI probability scores for each participant were calculated from feature vectors in the test sample of the outer loop of each cross‐validation fold. Here, an MCI probability score of 0 represented a normal aging (i.e., control) subject, while 1 represented a subject diagnosed as MCI.

### 2.10 Classification performance

In order to establish each model's ability to detect MCI within the RS cohort, predictions of each classification model were quantitatively compared using receiver‐operating characteristic (ROC) statistics. Predictions (values between 0 and 1) were compared with the actual diagnosis (0 = control, 1 = AD/MCI) at increasing probability thresholds. The area under the ROC curve (AUC) was calculated as a threshold‐independent measure of classification performance insensitive to the distribution of each patient group (Fawcett, 2006). The optimal operating point on the ROC curve (highest balanced accuracy) was used to calculate measures of accuracy, sensitivity, and specificity under equal class distribution, and equal false positive and false negative prediction penalty assumptions.

### 2.11 Statistical analysis

Demographic group differences between age, MMSE, and cognitive test scores were assessed using two‐tailed Wilcoxon‐rank sum tests. Gender and center distributions were assessed with *χ* ^2^ tests. To determine whether classification models performed differently for MCI detection, ROC curves were compared using a bootstrap percentile method for paired AUCs (Hanley & McNeil, 1983; Robin et al., 2011; two‐tailed, *N* = 5,000; single measure vs. multiparametric combination: one‐tailed). To determine whether a model performed better than chance, AUC values of each model were compared against chance level using permutation tests with maximum statistic method for family‐wise error correction (Winkler, Ridgway, Douaud, Nichols, & Smith, 2016). The calculated probability scores of each model were compared for MCI‐control contrasts using two‐tailed Wilcoxon‐rank sum tests, after being offset corrected by subtracting for each model the global minimal probability score from the calculated probability score. To determine whether overlap in probability scores differed for the evaluated classification models, calculated scores were compared using permutation tests and subsequently adjusted for multiple comparisons using Bonferroni correction (*N* = 5,000, one‐tailed). Statistical difference was considered at *p* < 0.05 for all tests.

All statistical analyses were implemented in R (R version: 3.2.3, R Core Team, 2014) using the glmnet (R version: 2.05), ROCR (R version: 1.0‐7), pROC (R version: 1.9.1), and caret (R version: 6‐0‐70) packages.

## 3 RESULTS

### 3.1 Demographics

For this study, 665 subjects of the RS cohort were included. Seventeen subjects were excluded from our analysis due to diffusion MRI acquisition artifacts that included large motion or eddy‐current induced artifacts (*N* = 4) or due to unresolvable postprocessing‐related artifacts (*N* = 13). Cognitive assessment scoring and MRI were on average conducted within 1.02 ± 0.46 years. Cognitive assessments diagnosed 48 subjects as mild cognitively impaired and 617 as cognitively normal (Table 1). Twenty‐three MCI subjects had substantial deficiencies in memory performance and were hence diagnosed as amnestic MCI. The remaining 25 MCI subjects were considered nonamnestic MCI (Table 2). The AD‐model, mild‐AD‐model, and moderate‐AD‐model were trained using feature vectors of the AD‐cohort. Compared with the RS cohort, subjects of the AD cohort were older and MMSE scores of AD patients (MMSE = 20 ± 5 [mean ± standard deviation]) were lower than MCI subjects of the RS cohort (MMSE = 26 ± 2, *p* < 0.001; Table 1).

**Table 2:** Demographics of amnestic MCI, nonamnestic MCI, and control subjects of the RS cohort

		RS cohort		
		Amnestic MCI	Nonamnestic MCI	Control
*N*		23	25	617
Age (mean ± *SD*)		69.9 ± 7.6	67.8 ± 5.4	67.3 ± 5.2
Female gender (%)		8 (34.8)	15 (60.0)	298 (51.7)
MMSE		27 [25–28]**	28 [26–29]*	28 [27–29]
Memory				
(median [iqr])	WLT im	7 [6–8]**	12 [10–15]§	14 [11–17]
	WLT delay	3 [2–4]**	6 [5–9]§	7 [6–9]
Information processing speed				
(median [iqr])	Stroop I	18.9 [16.9–20.7]	23.1 [19.4–29.7]** §	16.8 [15.0–18.3]
	Stroop II	24.8 [23.0–27.1]*	27.8 [25.5–31.0]**	22.4 [20.2–24.9]
	LDST	28 [22–30]*	23 [19–27]**	30 [26–35]
Executive functioning				
(median [iqr])	VFT	18 [16–21]**	16 [14–22]**	22 [19–26]
	Stroop III	62.6 [49.0–89.2]**	67.6 [55.7–97.0]**	46.2 [39.2–54.2]

delay: delayed recall; im: immediate recall; iqr: inter‐quartile range; LDST: letter digit substitution task; MCI: mild cognitive impairment; MMSE: mini‐mental state examination; *SD*: standard deviation; Stroop I: Stroop reading subtask; Stroop II: Stroop color‐naming subtask; Stroop III: Stroop interference subtask; VFT: verbal fluency test; WLT: 15‐word verbal learning test.

* Versus control subjects *p* < 0.05.

** Versus control subjects, *p* < 0.001.

§ Versus amnestic MCI subjects, *p* < 0.001.

### 3.2 AD‐model

**Figure 1:**
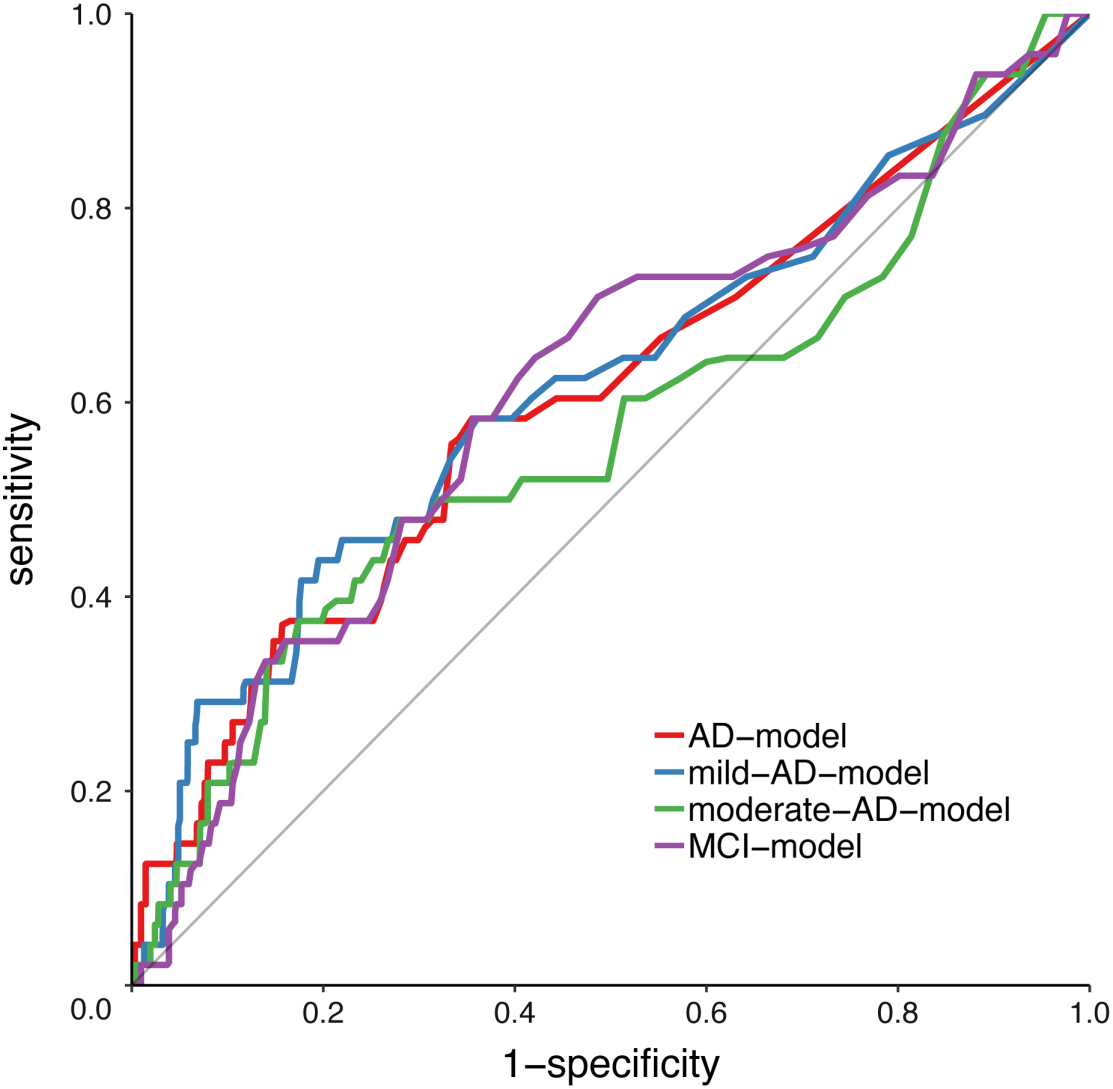
Receiver‐operating curves of MCI versus control classifications within the RS cohort. Classifications were obtained by training an AD versus control classification model using the AD cohort and subsequently applying it within the RS cohort (AD‐model). Mild‐AD‐model and moderate‐AD‐model classifications were calculated similarly to the AD‐model but respectively included mild‐AD patients (MMSE > 20) or moderate‐AD patients (MMSE ≤ 20) only. Finally, MCI versus control classifications were obtained through 10‐fold nested cross‐validation within the RS cohort (MCI‐model). Mean AUC values of classifications within the RS cohort were comparable (AD‐model: 0.600, mild‐AD‐model: 0.619; moderate‐AD‐model: 0.567; MCI‐model: 0.611 [Table 3]). Only classifications with the AD‐model (*p* = 0.025), mild‐AD‐model (*p* = 0.008)*,* and the MCI‐model (*p* = 0.014) were significantly better than chance level. The diagonal line represents random classification performance [Color figure can be viewed at http://wileyonlinelibrary.com]

**Figure 2:**
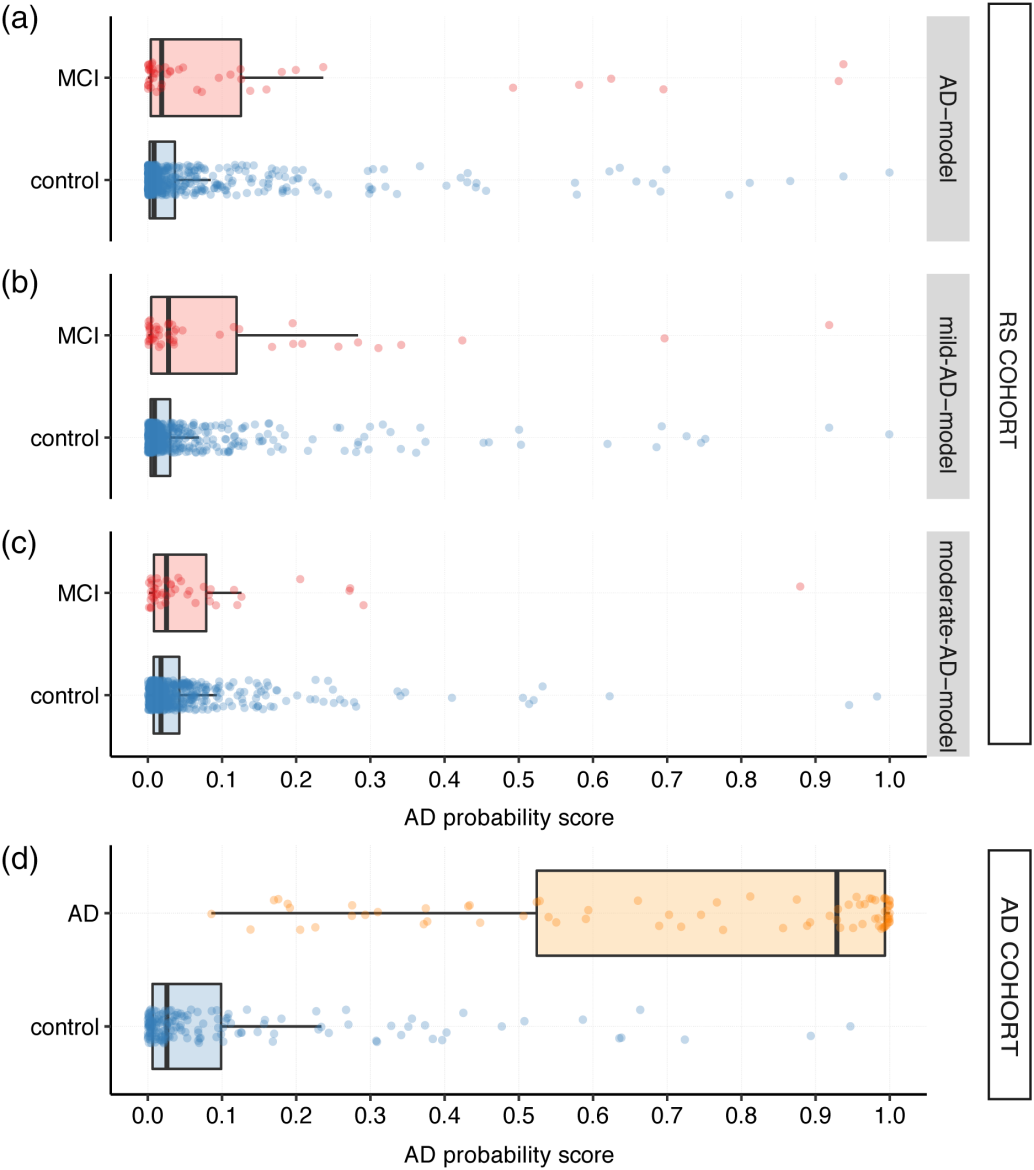
Box‐ and scatter plots of AD probability scores—ranging from control (0.0) to AD patient (1.0)—of each RS cohort subject as calculated with the AD‐model (a), mild‐AD‐model (b), or moderate‐AD‐model (c). AD probability scores calculated with the AD‐model (a) resulted from training an AD versus control classification model with all AD cohort subjects and subsequently applying it to subjects of the RS cohort. AD probability scores obtained with the mild‐AD‐model (b) were similarly calculated, but were trained with MRI measures of mild‐AD patients (MMSE > 20) and control subjects only, whereas AD probability scores of the moderate‐AD‐model (c) were calculated with MRI measures of moderate‐AD patients (MMSE ≤ 20) and control subjects only. AD‐model‐based probability scores from each subject in the AD cohort were added for reference (d). Within the RS cohort, mean AD probability scores for MCI subjects were higher than control subjects for classifications with the mild‐AD‐model (b, *p* = 0.047), but not for classifications with the AD‐model (a, *p* = 0.140) or moderate‐AD‐model (c, *p* = 0.870). Compared with scores within the AD cohort (d), AD probability scores within the RS cohort were lower and overlapped more between MCI and control subjects for the AD‐model (a, *p* = 0.002), mild‐AD model (b, *p* = 0.002), and moderate‐AD model (c, *p* = 0.002). For visual purposes, AD probability scores were offset adjusted by for each model subtracting each model's calculated minimal score from each subject's individual score [Color figure can be viewed at http://wileyonlinelibrary.com]

Optimization using single measure cross‐validation within the AD cohort revealed highest AUC values for single measure models either based on GMD‐ (0.925 [0.913–0.933] (mean AUC [min‐max]) or MD‐derived features (0.859 [0.838–0.872]). Yet, a classification model that included all MRI measures (AUC = 0.962 [0.948–0.974])) outperformed all single measure models (Supporting Information Table S2). This AD‐model was subsequently applied to the RS cohort for MCI detection. ROC analysis of the AD‐model resulted in mean AUC of 0.600, which was significantly higher than chance level (*p* = 0.025; Figure 1, Table 3). Overall, AD probability scores of MCI subjects (0.019 [0.004–0.13] (median [inter‐quartile range])) were comparable with control subjects (0.008 [0.002–0.036], *p* = 0.14) and overlapped more than the cross‐validated predictions within the AD cohort (*p* = 0.002, Figure 2a,e). AD probability scores of amnestic and nonamnestic MCI subjects were not different (*p* = 1.0).

**Table 3:** Classification performance values of the AD, mild‐AD, moderate‐AD, and MCI classification models within the RS cohort

Model	Measure	AUC	Min–max	Sensitivity	Specificity	Accuracy
AD	Multiparametric	0.600*	0.572–0.631	0.556	0.647	0.641
Mild‐AD	Multiparametric	0.619*	0.587–0.651	0.594	0.658	0.653
Moderate‐AD	Multiparametric	0.567	0.549–0.591	0.533	0.621	0.615
MCI	Multiparametric	0.611*	0.577–0.644	0.628	0.615	0.616

Mean, minimum, and maximum area under the ROC curve (AUC) after 100 classification repetitions. Classifications with the AD‐, mild‐AD‐, and moderate‐AD‐models resulted from 100 times repeated training on the AD cohort and applying it to the RS cohort. The MCI‐model resulted from 100 times repeated, 10‐fold nested cross‐validations using RS cohort data. Mean sensitivity, specificity, and accuracy were calculated at the optimal operating point on the ROC curve. DGMV: deep gray matter volumes; FA: fractional anisotropy; GMD: gray matter density; MD: mean diffusivity; Multiparametric: classification model including GMD, DGMV, WMD, FA, and MD; WMD: white matter density.

* Significantly higher than random classification, *p* < 0.05.

### 3.3 Mild‐AD‐model

Optimization of the mild‐AD‐model using cross‐validation in the AD cohort revealed highest AUC values of a classification model that included all MRI measures (mean AUC = 0.944 [0.913–0.959]). This multiparametric mild‐AD model outperformed all single MRI measure models, except GMD (0.896 [0.872–0.914], *p* = 0.07; Supporting Information Table S3). This mild‐AD‐model was subsequently applied to the RS cohort. AUC values of the mild‐AD‐model outperformed random chance classification (mean AUC = 0.619, *p* = 0.008) and were similar to AUC values of the AD‐model (*p* = 1.0; Figure 1, Table 3). The individual mild‐AD probability scores of MCI subjects (0.028 [0.005–0.12]) were higher than control subjects (0.009 [0.004–0.030], *p* = 0.047; Figure 2b), but did not differ between amnestic and nonamnestic MCI subjects (*p* = 1.0).

### 3.4 Moderate‐AD‐model

In agreement with the AD‐model and mild‐AD model, optimization of the moderate‐AD‐model using cross‐validation in the AD cohort, revealed highest AUC values for a classification model that included all features (mean AUC = 0.914 [0.884–0.935]; Supporting Information Table S4). This multiparametric moderate‐AD model outperformed single MRI measure models that included either DGMV (0.821 [0.788–0.835], *p* = 0.004), WMD (0.829 [0.811–0.844], *p* = 0.02), or FA (0.793 [0.763–0.817], *p* = 0.006) features only. This moderate‐AD‐model was subsequently applied to the RS cohort. AUC values of the moderate‐AD‐model (mean AUC = 0.567 [0.549–0.591]) were comparable to the AD‐model (*p* = 0.44; Figure 1, Table 3) or mild‐AD‐model (*p* = 0.26), but were not better than random chance classifications (*p* = 0.15). The individual moderate‐AD probability scores of MCI subjects (0.025 [0.008–0.079]) were similar to control subjects (0.018 [0.008–0.042], *p* = 0.87; Figure 2c) and did not differ between amnestic and nonamnestic MCI subjects (*p* = 1.0).

### 3.5 MCI‐model

**Figure 3:**
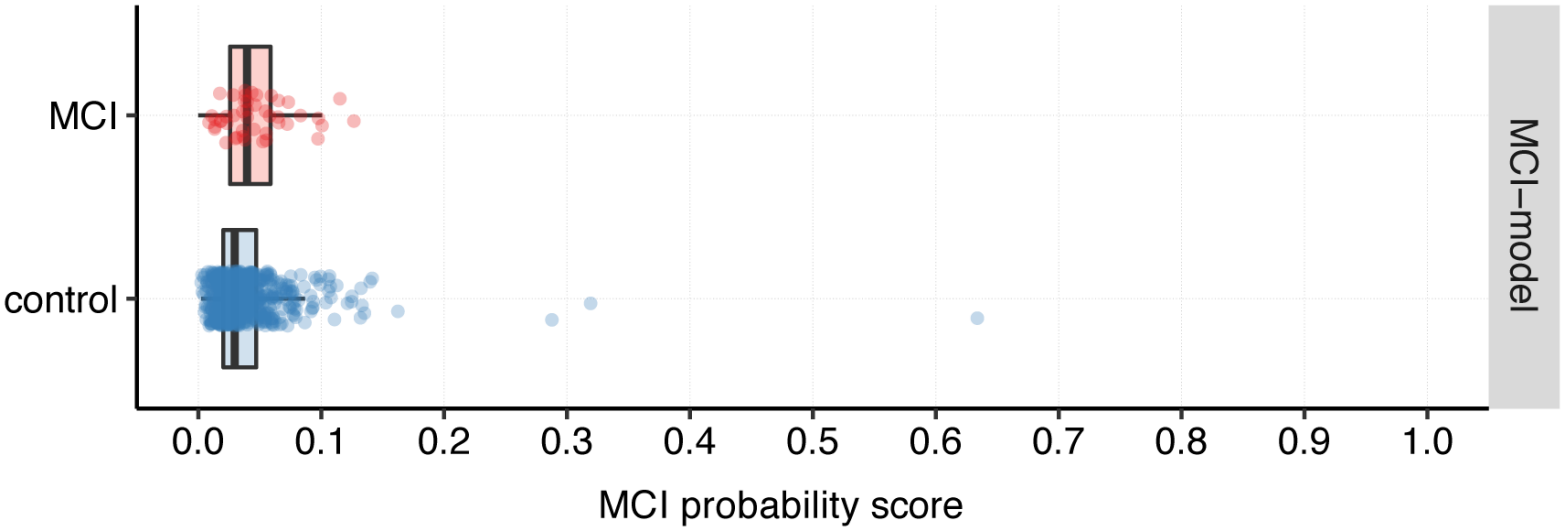
Box‐ and scatter plots of MCI probability score—ranging from control (0.0) to MCI (1.0) subject—of each RS cohort subject as calculated with the MCI‐model. Mean MCI probability scores for MCI subjects were slightly higher than control subjects (*p* = 0.060), but scores were lower and overlapped more than AD probability scores in the AD cohort (*p* = 0.002, Figure 2d). For visual purposes, MCI probability scores were offset adjusted by subtracting the MCI model's minimal score from each subject's individual score [Color figure can be viewed at http://wileyonlinelibrary.com]

Cross‐validation within RS cohort revealed highest classification performance rates for a model that included all MRI measures (mean AUC = 0.611 [0.577–0.644]; Supporting Information Table S5). This model outperformed random chance classifications (*p* = 0.014; Figure 1, Table 3) and was more accurate than classifications using WMD (*p* = 0.011) measures only (Supporting Information Table S5). Classification performance values were however not different from those of the AD‐model (*p =* 1.0), mild‐AD‐model (*p* = 1.0), or the moderate‐AD‐model (*p* = 1.0). On a group‐level, MCI probability scores of MCI subjects (0.040 [0.026–0.059]) were slightly higher than control subjects (0.030 [0.020–0.047], *p* = 0.060; Figure 3), but overlapped more than the AD probability scores of cross‐validated predictions within the AD cohort (*p* = 0.002, Figures 2d and 3). MCI probability scores between amnestic and nonamnestic MCI subjects were furthermore not different (*p* = 0.78).

## 4 DISCUSSION

In this study, we determined the generalizability of MRI‐based classification probability scores as an auxiliary tool for single subject detection of MCI in a population‐based cohort. We compared the classification performance of AD classification models, trained using a separate clinical AD cohort, with an MCI‐model, cross‐validated directly on the population‐based cohort, to detect MCI within a population‐based cohort. We found that performance rates were comparable between AD‐ and MCI‐models for the detection of MCI. AD‐, mild‐AD‐, and MCI‐models outperformed random chance classification. However, only probability scores of MCI subjects calculated with the mild‐AD‐model were significantly higher than cognitively normal subjects. Furthermore, classification rates were unequivocally low and classification probability scores overlapped significantly more than classification probability scores calculated in the clinical cohort.

Previous MRI‐based classification methods have been heralded as promising tools for accurate classification of AD (Bouts et al., 2018; Bron et al., 2016; Schouten et al., 2016), MCI (Cui et al., 2012; Eskildsen et al., 2013), or to differentiate between MCI subjects likely to develop dementia due to AD or those that do not progress (Adaszewski et al., 2013; Arbabshirani, Plis, Sui, & Calhoun, 2016; Eskildsen et al., 2013; Misra et al., 2009; Rathore et al., 2017). These studies generally aimed to maximize classification performance by using sparse, carefully selected clinical samples. Despite obvious merit in maximizing classification accuracy by creating time‐homogenized models (Eskildsen et al., 2013), or subject‐homogenized groups for training (Mendelson, Zuluaga, Lorenzi, Hutton, & Ourselin, 2017; Rathore et al., 2017), a priori selecting the proper model for a specific subject is difficult to achieve in practice and consequently may result in distorted estimations of disease severity (Mendelson et al., 2017). In this study, we used previously formulated multiparametric AD detection models (Bouts et al., 2018; Schouten et al., 2016) trained with a carefully assembled AD cohort to determine whether such a model can be used to accurately detect MCI individuals within a heterogenous, nonclinical population. Similar to this study, these models showed high classification performance in different clinical cohorts with AUC of around 0.94 (Bouts et al., 2018; Schouten et al., 2016). However, when translated to our population‐based cohort, we did not find that these models were effective in accurately detecting MCI. The AD‐model performed better than random chance in differentiating MCI from normal aging subjects, but classification performance rates were substantially lower than those observed in smaller (clinical) cohorts (Arbabshirani et al., 2016; Cui et al., 2012; Rathore et al., 2017). This is in line with previous work that used structural MRI data from a clinical cohort to determine diagnostic accuracies of a general AD classifier at different times prior to AD conversion (Adaszewski et al., 2013). While MCI converters were detected above chance level as early as 4 years prior to disease onset, accuracies were nevertheless low. Furthermore, we observed that MCI detection with the moderate‐AD‐model were below chance‐level performance and probability scores of MCI subjects were only significantly higher than normal aging subjects when calculated with the mild‐AD‐model. This agrees with perceptions that brain regions involved in early stage AD detection may better match those of MCI subjects than those regions considered relevant for the detection of more progressed AD patients (Adaszewski et al., 2013) and may allude to the fact that patient heterogeneity may have a strong influence on classification performance (Adaszewski et al., 2013; Eskildsen et al., 2013; Rathore et al., 2017).

Remarkably, classification performance of the dedicated MCI‐model did not improve over those of the AD‐models. Classification performance rates of the MCI‐model may have been biased by using the imbalanced RS cohort for both training and testing. While the other models used a separate clinical cohort for training. Nevertheless, it was previously observed that MCI detection models that used DTI‐derived measures (Dyrba et al., 2015) or combinations with measures of GM atrophy were best for the detection of MCI (Cui et al., 2012; Fan et al., 2008) or AD (Bron et al., 2016; Rathore et al., 2017; Schouten et al., 2016). We also found that only those models that either used DTI‐derived measures of impaired WM integrity or combined these with measures of GM atrophy were better than chance for MCI detection within the RS cohort. Nevertheless, all models resulted in similarly moderate classification performance values that were far from set criteria for acceptable detection (Bachurin et al., 2018; Thies, Truschke, Morrison‐Bogorad, & Hodes, 1998). Despite previously elucidated group‐wise differences (De Bruijn et al., 2014; Wang et al., 2017), it may therefore be that structural MRI‐ and DTI‐derived measures are not sufficiently sensitive for reliable MRI‐based single subject MCI detection.

In our study, we used a modified, data‐driven MCI diagnosis that was based on existing clinical criteria (Jack et al., 2018; Petersen et al., 1999) and was previously established and employed within a larger part of the Rotterdam study (Adams et al., 2015; De Bruijn et al., 2014). These criteria identified 7% of the included participants as MCI, which agrees well with MCI prevalence estimates of around 5–22% within the general population (Hanninen, Hallikainen, Tuomainen, Vanhanen, & Soininen, 2002; Lopez et al., 2003). Nevertheless, although this diagnosis may have facilitated early detection and exposed group‐wise differences (De Bruijn et al., 2014), it may have challenged detection on an individual level. Cognitive abnormalities and MRI‐detectable differences between MCI and normal aging are likely more heterogeneous (Haller et al., 2013) and less conspicuous than observed in clinical MCI cohorts (Adaszewski et al., 2013; De Bruijn et al., 2014). It could also suggest that our MCI subjects were still far from disease onset or may not progress to dementia at all (Roberts et al., 2014). While our multidisciplinary, multicenter team carefully followed contemporary guidelines for AD (McKhann et al., 1984) and MCI (De Bruijn et al., 2014; Jack et al., 2018; Petersen et al., 1999) diagnosis, diagnosis remains provisional. Postmortem pathological data to confirm AD diagnosis were unavailable and MCI represents an intermediate stage for which outcome remains uncertain (Petersen, 2011; Roberts et al., 2014; Visser, Kester, Jolles, & Verhey, 2006). We combined amnestic MCI and nonamnestic MCI subjects to maximize our MCI sample. We did not observe differences in probability scores of amnestic or nonamnestic MCI subjects. Nevertheless, heterogeneity in the underlying etiology of amnestic and nonamnestic MCI may have further mitigated classification performance (Guan et al., 2017). Especially at longer follow‐up times, amnestic MCI patients are more likely to develop AD‐like atrophy patterns and are more likely to convert to AD (Roberts et al., 2014; Visser et al., 2006). To establish disease trajectories, longer follow‐up times are needed which were unavailable for this study (Roberts et al., 2014).

In our analysis, we took several steps to reduce center related discrepancies, reduce classification bias, and maximize the generalizability of our results. First, MRI related differences such as field‐strength (1.5 T vs. 3 T), head‐coil, and MRI sequence settings were addressed prior to model training by estimating linear correction factors using alternating subgroups of control subjects. Control subjects were used to make sure that possible subject and scanner interactions were principally related to normal aging rather than disease induced patterns (Abdulkadir et al., 2011; Dukart et al., 2011). Second, for classification analysis we used a previously introduced AD‐model that was validated on the same cohort with similar results (Schouten et al., 2016). This model was based on regularized regression to construct stable classification probability estimates and to accommodate selection of relevant features despite high dimensionality and collinearity of our data. Classifications were repeated to reduce variance in classification performance evaluations. Nested cross‐validations were used to furthermore ensure unbiased regression parameter optimization (Mendelson et al., 2017; Varma & Simon, 2006; Varoquaux, 2018). Thirdly, although of great interest, we refrained from biological interpretation of the model's parameters and weights. The trained models rely heavily on both random and nonrandom class differences and consequently cannot reliably differentiate between true or random class differences (Varoquaux et al., 2017). Additionally, nonzero weights of the selected features are mutually dependent and may originate from sources statistically independent of disease‐related brain regions (Haufe et al., 2014).

In our work, we focused on establishing whether a previously outlined multiparametric MRI‐based AD detection approach (Bouts et al., 2018; Schouten et al., 2016) could be applied as an additional tool for robust MCI detection. We found that this translation may not be straightforward. Other works did, however, show promise in identifying those subjects more likely to convert to dementia using amnestic MCI subjects and dedicated models for training (Cui et al., 2012; Wang et al., 2017). It may therefore suggest that more tailored approaches that focus on MCI‐specific biomarkers are necessary to fully capture the subtle complexities of neurodegenerative processes underlying early stage MCI or dementia. It is however questionable whether MRI‐based algorithms that only incorporate structural or diffusion MRI‐derived measures can fully capture this complexity (Jack et al., 2018). Classification performance rates of the MCI‐model did not improve over those of the AD‐model or mild‐AD‐model. The incorporation of additional prior, biological knowledge (Rathore et al., 2017), or other information derived from imaging‐ and nonimaging biomarkers such as cerebrovascular status (De Bruijn et al., 2014), the load (De Bruijn et al., 2014; Dong et al., 2017; Fan et al., 2008), or location of white matter hyperintensities (McAleese et al., 2017), cerebral blood flow (Bron et al., 2016), resting‐state functional MRI (de Vos et al., 2017; Schouten et al., 2016), PET‐derived biomarkers (Dukart et al., 2013; Li et al., 2014), or additional cognitive assessment scores including measures of cognitive reserve (Allegri et al., 2010; Moradi et al., 2015; Vieira, Pinaya, & Mechelli, 2017; Wang et al., 2017) may further augment classification accuracy without increasing diagnostic complexity. While cognitive assessment scores would most likely provide a valuable contribution to the detection of MCI (Moradi et al., 2015; Wang et al., 2017), we did not consider these for this study. Cognitive assessment scores were used to establish our MCI diagnosis and would most likely bias classification performance results and provide a skewed perception of the contribution of each modality to the classification result. Other machine learning methods that do not need a priori feature generation and selection such as deep learning‐based methods (Bowles, Gunn, & Hammers, 2018; Vieira et al., 2017), or methods that exploit longitudinal (McEvoy et al., 2011), or augmented data (Bowles et al., 2018; Li et al., 2014) may furthermore provide adept means to improve detection.

## 5 CONCLUSION

We investigated multiparametric MRI‐based classifiers, that were trained to identify AD‐like patterns, in their ability to detect MCI within a community‐dwelling cohort. We did not find that multiparametric MRI‐based classification probability scores were suitable as an auxiliary tool for accurate MCI detection in a general population. Our findings suggest that MRI‐based algorithms that are effective in clinical cohorts may not straightforwardly translate to MCI detection in a population‐based cohort. More tailored solutions, that integrate multiple MCI‐specific imaging and nonimaging biomarkers, may be warranted for robust MCI detection within the general population.

## CONFLICT OF INTEREST

The authors report no conflict of interest.

## Supporting information

Appendix S1: Supplementary Information
