# Supplementary material for: Detection of mild cognitive impairment in a community‐dwelling population using quantitative, multiparametric MRI‐based classification
Source: Hum Brain Mapp. 2019 Feb 25;40(9):2711–22. doi: 10.1002/hbm.24554 (PMC6563478; doi:10.1002/hbm.24554)
Supplement: Supplementary file 1 — Appendix S1: Supplementary Information [file HBM-40-2711-s001.doc]

Supporting information to:

*Detection of mild cognitive impairment in a community-dwelling population using quantitative, multiparametric MRI-based classification*

# Supplementary Tables & Figures

**Supporting Information** **Table S1.** Demographics of Control, mild-AD (MMSE > 20), and moderate-AD subjects (MMSE<=20) within the AD cohort

|  | **AD cohort** | | |  |  |
| --- | --- | --- | --- | --- | --- |
|  | **Control** |  | **Mild-AD** |  | **Moderate-AD** |
| N | 173 |  | 39 |  | 38 |
| Age (mean ± sd) | 66.1 ± 8.7 |  | 70.3 ± 7.9* |  | 66.9±9.1 |
| Female gender (%) | 99 (57.2) |  | 22 (56.4) |  | 24 (63.2) |
| Disease duration (months) |  |  | 22.0 ± 15.7 |  | 30.9±30.7 |
| MMSE (mean±sd) | 27.5 ± 1.8 |  | 24.2 ± 2.1** |  | 16.6±2.7**§ |

MMSE: mini-mental state examination, AD: Alzheimer’s disease, sd: standard deviation. * versus Control subjects, *P*<0.05; ** versus Control subjects, *P*<0.01; § versus Mild-AD subjects, *P*<0.01.

**Supporting Information** **Table S2.** Classification performance values of the AD-model after 100 repetitions of 10-fold nested cross-validation within the AD cohort.

| **Model** | **Measure** | **AUC** | **Min - Max** | **Sensitivity** | **Specificity** | **Accuracy** |
| --- | --- | --- | --- | --- | --- | --- |
| AD | GMD | 0.925*^#^ | 0.913 - 0.933 | 0.843 | 0.815 | 0.824 |
| AD | DGMV | 0.819*^#§^ | 0.805 - 0.833 | 0.709 | 0.684 | 0.692 |
| AD | WM | 0.834*^#§^ | 0.815 - 0.846 | 0.751 | 0.818 | 0.798 |
| AD | FA | 0.810*^#§^ | 0.795 - 0.824 | 0.733 | 0.779 | 0.764 |
| AD | MD | 0.859*^#^ | 0.838 - 0.872 | 0.806 | 0.818 | 0.815 |
| **AD** | **Multiparametric** | **0.962*** | **0.948 - 0.974** | **0.897** | **0.863** | **0.873** |

Mean, minimum, and maximum area under the ROC curve (AUC) after 100 nested cross-validation repetitions using AD cohort data. Mean sensitivity, specificity, and accuracy were calculated at the optimal operating point on the ROC curve. Bold: best performing classification model after cross-validation. GMD: gray matter density, DGMV: subcortical gray matter volumes, WMD: white matter density, FA: fractional anisotropy, MD: mean diffusivity. Multiparametric: classification model including GMD, DGMV, WMD, FA, and MD. * significantly higher than random classification, *P*<0.05. # significantly lower than multiparametric AD-model, *P*<0.05. § significantly lower than GMD-based AD-model, *P*<0.05.

**Supporting Information** **Table S3.** Classification performance values of the mild-AD-model after 100 repetitions of 10-fold nested cross-validation within the AD cohort.

| **Model** | **Measure** | **AUC** | **Min - Max** | **Sensitivity** | **Specificity** | **Accuracy** |
| --- | --- | --- | --- | --- | --- | --- |
| mild-AD | GMD | 0.896* | 0.872 - 0.914 | 0.798 | 0.837 | 0.830 |
| mild-AD | DGMV | 0.811*^#^ | 0.789 - 0.831 | 0.811 | 0.686 | 0.709 |
| mild-AD | WM | 0.802*^#^ | 0.760 - 0.831 | 0.739 | 0.737 | 0.737 |
| mild-AD | FA | 0.795*^#^ | 0.767 - 0.818 | 0.729 | 0.787 | 0.776 |
| mild-AD | MD | 0.818*^#^ | 0.793 - 0.843 | 0.778 | 0.764 | 0.766 |
| mild-AD | **Multiparametric** | **0.944*** | **0.913 - 0.959** | **0.872** | **0.884** | **0.882** |

Mean, minimum, and maximum area under the ROC curve (AUC) after 100 nested cross-validation repetitions using AD cohort data. Mean sensitivity, specificity, and accuracy were calculated at the optimal operating point on the ROC curve. Bold: best performing classification model after cross-validation. Sensitivity, specificity, and accuracy were calculated at the optimal operating point on the ROC curve. GMD: gray matter density, DGMV: subcortical gray matter volumes, WMD: white matter density, FA: fractional anisotropy, MD: mean diffusivity. Multiparametric: classification model including GMD, DGMV, WMD, FA, and MD. *significantly higher than random classification, *P*<0.05. # significantly lower than multiparametric mild-AD-model, *P*<0.05

**Supporting Information** **Table S4.** Classification performance values of the moderate-AD-model after 100 repetitions of 10-fold nested cross-validation within the AD cohort.

| **Model** | **Measure** | **AUC** | **Min - Max** | **Sensitivity** | **Specificity** | **Accuracy** |
| --- | --- | --- | --- | --- | --- | --- |
| MCI | GMD | 0.906* | 0.883 - 0.926 | 0.831 | 0.837 | 0.836 |
| MCI | DGMV | 0.821*^#^ | 0.788 - 0.835 | 0.825 | 0.760 | 0.772 |
| MCI | WM | 0.829*^#^ | 0.811 - 0.844 | 0.777 | 0.846 | 0.833 |
| MCI | FA | 0.793*^#^ | 0.763 - 0.817 | 0.717 | 0.788 | 0.776 |
| MCI | MD | 0.878* | 0.864 - 0.894 | 0.826 | 0.821 | 0.822 |
| **MCI** | **Multiparametric** | **0.914*** | **0.884 - 0.935** | **0.846** | **0.877** | **0.871** |

Mean, minimum, and maximum area under the ROC curve (AUC) after 100 nested cross-validation repetitions using RS cohort data. Mean sensitivity, specificity, and accuracy were calculated at the optimal operating point on the ROC curve. Bold: best performing classification model after cross-validation. GMD: gray matter density, DGMV: subcortical gray matter volumes, WMD: white matter density, FA: fractional anisotropy, MD: mean diffusivity. Multiparametric: classification model including GMD, DGMV, WMD, FA, and MD. * significantly higher than random classification, *P*<0.05. # significantly lower than multiparametric moderate-AD-model, *P*<0.05.

**Supporting Information** **Table S5.** Classification performance values of the MCI-model after 100 repetitions of 10-fold nested cross-validation within the RS cohort.

| **Model** | **Measure** | **AUC** | **Min - Max** | **Sensitivity** | **Specificity** | **Accuracy** |
| --- | --- | --- | --- | --- | --- | --- |
| MCI | GMD | 0.533 | 0.487 - 0.564 | 0.473 | 0.630 | 0.619 |
| MCI | DGMV | 0.500^#^ | 0.433 - 0.551 | 0.496 | 0.536 | 0.533 |
| MCI | WM | 0.531^#^ | 0.484 - 0.581 | 0.518 | 0.590 | 0.585 |
| MCI | FA | 0.607* | 0.555 - 0.640 | 0.612 | 0.627 | 0.626 |
| MCI | MD | 0.589* | 0.533 - 0.631 | 0.560 | 0.639 | 0.633 |
| **MCI** | **Multiparametric** | **0.611*** | **0.577 - 0.644** | **0.630** | **0.615** | **0.616** |

Mean, minimum, and maximum area under the ROC curve (AUC) after 100 nested cross-validation repetitions using RS cohort data. Mean sensitivity, specificity, and accuracy were calculated at the optimal operating point on the ROC curve. Bold: best performing classification model after cross-validation. GMD: gray matter density, DGMV: subcortical gray matter volumes, WMD: white matter density, FA: fractional anisotropy, MD: mean diffusivity. Multiparametric: classification model including GMD, DGMV, WMD, FA, and MD. * significantly higher than random classification, *P*<0.05. # significantly lower than multiparametric MCI model, *P*<0.05.

# Supporting Information Methods

## MRI

All 682 RS subjects were scanned on a 1.5T MRI scanner (GE Healthcare) with an 8-channel head coil. This protocol included an 3D T_1_-weighted image (repetition time (TR)=13.8 ms, echo time (TE)=2.8 ms, inversion time (TI)=400, flip angle (α)=20^o^, voxel dimensions= 1.6 mm^3^, slice thickness 0.8 mm after zero-padding) and diffusion MRI with maximum b-value was 1000 s/mm^2^ in 25 non-collinear directions (number of excitations (NEX)=1, TR=8000 ms, TE=120 ms, voxel-dimensions=3.3x2.2x3.5 mm^3^) and one volume was acquired without diffusion weighting (b-value=0 s/mm^2^).

All 250 subjects of the AD cohort were scanned on a 3T MRI scanner (TrioTim, Siemens) with a 12-channel head coil. The protocol included an 3D isotropic T1-weighted image (MPRAGE TR=1900 milliseconds (ms), TE=2.2ms, TI=900ms, α=9^o^, voxel dimensions=1x1x1 mm^3^) and diffusion MRI (b-value=1000 s/ mm^2^, 12 non-collinear directions, NEX = 4, TR=6700 ms, TE=95 ms, voxel dimensions=2x2x2.5 mm^3^).

## Image preprocessing

3DT_1_w images were non-uniformity corrected [Tustison et al., 2010] and parenchymal tissue signal was segmented from surrounding tissue using the brain extraction tool (BET) [Smith, 2002]. Images were then spatially aligned to the MNI152 1.0 mm^3^ T_1_ template (Montreal Neurological Institute, Canada) using non-linear registration procedures (For AD cohort: [Andersson et al., 2007], For RS cohort: [Klein et al., 2010]). Voxel-wise densities of GM, WM, and cerebrospinal fluid (CSF) were determined with the initial steps of the voxel-based morphometry pipeline of the Statistical Parametric Mapping toolbox (SPM8; Functional Imaging Laboratory, University College London, London, UK) [Friston et al., 2007]. Deep gray matter (DGMV) structures including the bilateral thalamus, caudate nucleus, putamen, globus pallidus, nucleus accumbens, amygdala, and left and right hippocampus were separately identified using a dedicated registration and segmentation procedure with default settings [Patenaude et al., 2011].

Diffusion MRI were first corrected for motion and eddy-current induced distortions. [Leemans and Jones, 2009] The corrected diffusion MRI and gradient vector directions were subsequently used to voxel-wise calculate measures of fractional anisotropy (FA), mean diffusivity (MD). Diffusion MRI and 3DT_1_-w images were linearly aligned using a boundary-based registration metric [Greve and Fischl, 2009]. Registrations to and from MNI were obtained by concatenating the T_1_ to MNI and diffusion MRI to T_1_ transformations. All registrations and segmentations were critically assessed visually and corrected if necessary.

## Feature extraction

The dedicated segmentations of DGMV, hippocampus, and amygdala were used to calculate the regional volumes normalized by total brain volume to compensate for individual differences in brain volume [de Vos et al., 2016]. The global GM and WM segmentations were used to calculate GMD (GMD) and WM densities (WMD). Two anatomical atlases were used to extract cortical GM and tract-specific WM features in each subject’s native space. The cortical Harvard–Oxford (HO) probabilistic anatomical brain atlas was split into left and right hemisphere identifying 96 GM regions that covered the entire cortical GM. These regions were used to calculate regional cortical gray matter densities (GMD) by voxel-wise weighting the GM segmentations by the probability (>25%) of a voxel being part of the brain atlas-derived region. The probabilistic Johns-Hopkins-University (JHU) white-matter tractography atlas was used to select 20 WM tracts [Hua et al., 2008]. Each tract was used to calculate the weighted WM densities (WMD) of that tract by weighting the WM segmentations by the atlas-derived probability (>25%) of a voxel being part of that specific tract. This way we affirmed the regions’ likelihood of being GM or WM without introducing bias resulting from conservatively selecting brain regions. This resulted in a feature vector of 96 cortical GMD values, 14 DGMV volumes, and 20 average WMD values per subject.

For DTI-based features, the standard FMRIB58_FA skeleton of the tract-based spatial statistics (TBSS) pipeline [Smith et al., 2007] was transformed into local space and thresholded at 0.2 to ensure values originated from WM tissue. FA and MD maps were upsampled to 1 mm^3^ isotropic voxels and subsequently values were voxel-wise projected on to the skeleton. Tract-specific weighted mean FA and MD values were obtained by weighting mean skeletonized values by the JHU atlas-derived probability (>25%) of a voxel being part of a specific tract. This resulted in 2*20 feature vectors of mean FA and MD values per subject.

## References

Andersson JLR, Jenkinson M, Smith S (2007): Non-linear registration, aka spatial normalisation. FMRIB Technial Report TR07JA2. Oxford Centre for Functional Magnetic Resonance Imaging of the Brain, Department of Clinical Neurology, Oxford University, Oxford, UK. Vol. 2.

Friston KJ, Ashburner J, Kiebel S, Nichols T, Penny WD (2007): Statistical parametric mapping : the analysis of funtional brain images. Elsevier/Academic Press.

Greve DN, Fischl B (2009): Accurate and robust brain image alignment using boundary-based registration. Neuroimage 48:63–72. http://www.ncbi.nlm.nih.gov/pubmed/19573611.

Hua K, Zhang J, Wakana S, Jiang H, Li X, Reich DS, Calabresi PA, Pekar JJ, van Zijl PCM, Mori S (2008): Tract probability maps in stereotaxic spaces: analyses of white matter anatomy and tract-specific quantification. Neuroimage 39:336–347. http://www.ncbi.nlm.nih.gov/pubmed/17931890.

Klein S, Staring M, Murphy K, Viergever MA, Pluim JP (2010): elastix: a toolbox for intensity-based medical image registration. IEEE TransMedImaging 29:196–205. pm:19923044.

Leemans A, Jones DK (2009): The B-matrix must be rotated when correcting for subject motion in DTI data. Magn Reson Med 61:1336–1349. http://www.ncbi.nlm.nih.gov/pubmed/19319973.

Patenaude B, Smith SM, Kennedy DN, Jenkinson M (2011): A Bayesian model of shape and appearance for subcortical brain segmentation. Neuroimage 56:907–922. http://www.ncbi.nlm.nih.gov/pubmed/21352927.

Smith SM (2002): Fast robust automated brain extraction. Hum Brain Mapp 17:143–155.

Smith SM, Johansen-Berg H, Jenkinson M, Rueckert D, Nichols TE, Miller KL, Robson MD, Jones DK, Klein JC, Bartsch AJ, Behrens TEJ (2007): Acquisition and voxelwise analysis of multi-subject diffusion data with tract-based spatial statistics. Nat Protoc 2:499–503. http://www.ncbi.nlm.nih.gov/pubmed/17406613.

Tustison NJ, Avants BB, Cook PA, Zheng Y, Egan A, Yushkevich PA, Gee JC (2010): N4ITK: improved N3 bias correction. IEEE Trans Med Imaging 29:1310–1320. http://www.ncbi.nlm.nih.gov/pubmed/20378467.

de Vos F, Schouten TM, Hafkemeijer A, Dopper EGP, van Swieten JC, de Rooij M, van der Grond J, Rombouts SARB (2016): Combining multiple anatomical MRI measures improves Alzheimer’s disease classification. Hum Brain Mapp 37:1920–1929. http://www.ncbi.nlm.nih.gov/pubmed/26915458.
